# Structure-Activity Association of Flavonoids in Lung Diseases

**DOI:** 10.3390/molecules19033570

**Published:** 2014-03-24

**Authors:** João Henrique G. Lago, Alessandra C. Toledo-Arruda, Márcia Mernak, Kaidu H. Barrosa, Milton A. Martins, Iolanda F. L. C. Tibério, Carla M. Prado

**Affiliations:** 1 Instituto de Ciências Ambientais, Químicas e Farmacêuticas, Universidade Federal de São Paulo, Diadema–SP 09972-270, Brazil; 2 Faculdade de Medicina, Universidade de São Paulo, São Paulo–SP 01246903, Brazil

**Keywords:** flavonoids, anti-inflammatory response, lung disease

## Abstract

Flavonoids are polyphenolic compounds classified into flavonols, flavones, flavanones, isoflavones, catechins, anthocyanidins, and chalcones according to their chemical structures. They are abundantly found in Nature and over 8,000 flavonoids have from different sources, mainly plant materials, have been described. Recently reports have shown the valuable effects of flavonoids as antiviral, anti-allergic, antiplatelet, antitumor, antioxidant, and anti-inflammatory agents and interest in these compounds has been increasing since they can be helpful to human health. Several mechanisms of action are involved in the biological properties of flavonoids such as free radical scavenging, transition metal ion chelation, activation of survival genes and signaling pathways, regulation of mitochondrial function and modulation of inflammatory responses. The anti-inflammatory effects of flavonoids have been described in a number of studies in the literature, but not frequently associated to respiratory disease. Thus, this review aims to discuss the effects of different flavonoids in the control of lung inflammation in some disorders such as asthma, lung emphysema and acute respiratory distress syndrome and the possible mechanisms of action, as well as establish some structure-activity relationships between this biological potential and chemical profile of these compounds.

## 1. Introduction

Flavonoids are cytoprotective compounds that are present in dietary plants and vegetables. The anti-inflammatory and antioxidant properties of flavonoids make them likely candidates for evaluation for the treatment of inflammatory diseases including pulmonary diseases [1,2,3,4]. In addition, flavonoids afford cellular protection by inhibiting enzymes involved in cell proliferation and modulating the expression of proteins associated with apoptosis [5]. In the current review, we focus on the anti-inflammatory activity of flavonoids with respect to respiratory illness and correlate the structural and functional relationship of these compounds. 

## 2. Chemistry and Occurrence of Flavonoids

**Figure 1:**
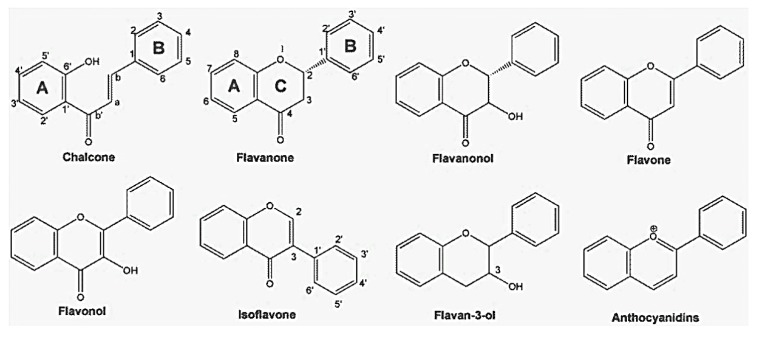
Classification of flavonoids based on their chemical structure.

**Figure 2:**
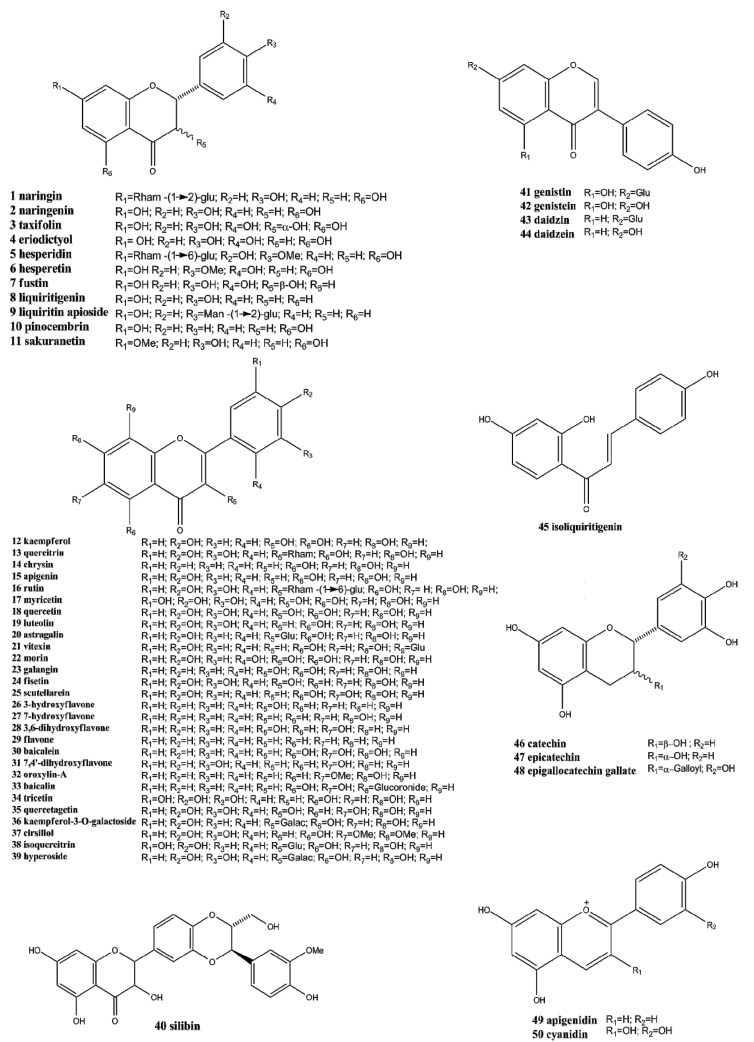
Chemical structures of flavonoids **1**–**50**.

Flavonoids, the most important class of phenolic compounds, are secondary metabolites produced by plants and are found in a non-glycosylated form (aglycone) or attached to a sugar molecule (glycoside) [1]. All flavonoids display a common structure of two aromatic rings connected to three carbon atoms (C_6_'C_3_'C_6_). These compounds frequently displayed hydroxylation at positions 5 and 7 and the B ring and oxidation at the 4'-, 3',4'-, or 3',4',5'- positions due to their biosynthesis routes [6]. Flavonoids are widely found in Nature and present an important chemical diversity mainly because of the variations in the positions of B and C rings and the degree of hydroxylation, oxidation and saturation, of C ring. Flavonoids are generally classified according to their chemical structures into chalcones, flavanones, flavanonols, flavones, flavonols, isoflavones, flavan-3-ols (or catechins), and anthocyanidins [7,8,9], as shown in Figure 1. Structures of the various flavonoids mentioned in this review are presented in Figure 2.

Chalcones, considered to be the precursors of flavonoids, consist of two aromatic rings connected by three carbons to form an α,β-unsaturated carbonyl system. After the C ring is closed to form a chromone unit, flavanones are formed. Flavanones display a carbonyl group in position C-4 as shown in the structures of naringin (**1**), naringenin (**2**), taxifolin (**3**), eriodictyol (**4**) and hesperidin (**5**). Oxidation at the C-3 position produces the flavonols kaempferol (**12**) and quercitrin (**13**). 

A double bond at C-2, formed through dehydration, produces flavones such as chrysin (**14**), apigenin (**15**) and rutin (**16**) as well as isoflavones such as genistin (**41**), genistein (**42**) daidzin (**43**), and daidzein (**44**). The action of reductases on flavonols produces flavan-3-ols such as catechin (**46**), epicatechin (**47**), and epigallocatechin gallate (**48**), and the colorful anthocyanidins, such as apigenidin (**49**) and cyanidin (**50**).

More than 8,000 different types of flavonoids have been described in Nature. These compounds, usually described as glycoside forms (e.g., glucosides, rhamnoglucosides, and rutinosides), are found in our daily diet in fruits, vegetables, grains, seeds, bark, herbs, roots, flowers, stems, and spices [7,10,11,12]. The consumption of flavonoids not only occurs through intake of vegetables, but also by drinking beverages such as wine, tea and coffee [13]. These polyphenols have a wide variety of physiological functions in plants raging from affecting plant pigmentation, flavor, growth and reproduction to providing an innate immunity and resistance against pathogens (bacterial, fungal and viral) and also protection against herbivores and insects [1]. Flavonoids are also involved in electron transport during photosynthesis, acting as an antioxidant against the effects of ultraviolet light [14,15]. In addition, a wide variety of commercially available drugs have had their active compounds discovered from naturally occurring products [16,17].

## 3. General Aspects and Structure-Activity of Flavonoids in the Oxidative Stress

Oxidative stress initiates a number of pathologic processes, including airway inflammation which is involved in the pathogenesis and also in exacerbation of pulmonary disease. The antioxidant defense mechanism includes enzymes such as superoxide dismutase, catalase and glutathione peroxidase. Flavonoids have been recognized as antioxidants because they protect against injuries caused by free radicals, and it has been suggested that flavones and flavan-3-ols are the most effective against reactive oxygen species [12]. The role of any particular phenolic antioxidant is directly associated with the capacity of the hydrogen radical donation from the phenolic group and the presence of an unpaired electron in the aromatic ring. 

The antioxidant activity of the non-glycosylated flavonoids is related to the number of hydroxyl groups present in the molecule [12]. However, the presence of a third hydroxyl group at the B ring at C-5 position on the structure of myricetin (**17**) does not increase its efficiency in comparison with quercetin (**18**). This suggests that the occurrence of three hydroxyl groups in the aromatic nucleus does not increase the efficiency of an antioxidant molecule. The presence of a hydroxyl group at the B ring of the chemical structure of kaempferol (**12**) could explain the low antioxidant activity of kaempferol (**12**), despite the presence of a conjugated double bond and the 3-OH group [18,19]. Other structural characteristic that influence the antioxidant activity are the 3'- and 4'-OH groups because the addition of hydroxyl groups to carbon atoms in the *ortho* position appear to further enhance the antioxidant capacity [19]. Several studies examining the activity [20,21,22,23] and structural characteristic of flavonoids have shown antioxidant and free radical scavenging properties. Three structurally important features are involved: (A) an *ortho*-dihydroxyl (catechol) group at the B ring, (B) a Δ^2^ double bond combined with a 4-oxo group in the C ring, and (C) hydroxyl groups at the C-3 and C-5 positions [24].

Additionally, evidence in the literature has showvn that non-glycosylated derivatives such as quercetin (**18**), luteolin (**19**), myricetin (**17**) and kaempferol (**12**) displayed a higher antioxidant capacity than the related flavonoids quercitrin (**13**), rutin (**16**) and astragalin (**20**) [25]. A previous study [26] demonstrated that quercetin glycosides have lower antioxidant activity than quercetin aglycone in an artificial membrane system, suggesting that glycosylation weakens declines the antioxidant effect of flavonoids. This reduction may be result of increased blockage of the phenolic groups responsible for removing radicals and metal chelating agents and perhaps to a decrease of accessibility of the membranes due to the large glycoside group. Higher oligomers have antioxidant potentials, and monomers have not as much of an effect [27].

Flavonols and flavones have similar structures but the lack of a 3-OH functional group in flavones is related to their low antioxidant activity. This statement is reinforced by a study [18] which describes the significant reduction of antioxidant activity (assessed by the Trolox equivalent antioxidant capacity—TEAC assay) of hyperoside (**39**), in which the 3-OH group in the C ring and the 3',4'-dihydroxy unit in the B ring are maintained. This study also proposes that the existence of a sugar moiety at position C-8 of the compound vitexin (**21**) significantly decreases antioxidant efficacy compared with the non-glycosylated derivative [18]. Other studies also show that substituting the 3-OH group with a methyl group decreases the activity of the glycosyl derivatives of quercetin (**18**) and kaempferol (**12**) against heat-induced oxidation by β-carotene and linoleic acid and affects the conformation of the molecule [28]. Moreover in a chemical scavenging study, Bors *et al.* [20] also showed that flavan-3-ols and flavonols are powerful antioxidants as a result of the presence of a 3-OH associated with the 3',4'-dihydroxyl (catechol) unit. Otherwise, the absence of the double bond decreases the antioxidant efficacy of flavanones (naringenin, **2**) in contrast to flavones (apigenin, **15**) and flavonols (quercetin, **18**). 

In addition, *in vivo*, certain flavonoids can remove superoxides directly, while others can remove the abundantly reactive peroxynitrite radicals [29,30,31]. Currently, studies show that the flavonoids quercetin (**18**), silibin (**40**) and luteolin (**19**) are the most potent inhibitors of xanthine oxidase [32]. Furthermore, flavonoids have the ability to chelate iron [33] and reduce the activation of the complement system thus reducing the adhesion of inflammatory cells to the endothelium [34], as shown using microvascular methods *in vitro* and *in vivo*. In addition, these compounds can inhibit the metabolism of arachidonic acid and reduce the peroxidase release, which in turn inhibits the production of reactive oxygen species by neutrophils by α1-antitrypsin activation [35]. Moreover, some flavonoids can chelate transition metal ions which are responsible for the production of reactive oxygen species and inhibit the lipoxygenase reaction. Zhu *et al*. [36] suggested that flavonoids also displayed an *in vivo* antioxidant capacity by the non-transition oxidation of the metal-dependent ions.

Flavonoids may also have antioxidant properties through the protection or improvement of endogenous antioxidants. Many of these compounds reduce oxidative stress by stimulating glutathione-S-transferase (GST), an enzyme that protects cells of the damage caused by free radicals by inducing oxidative stress resistance [37]. Because of this ability to reduce oxidation, flavonoids have been extensively tested in several studies of diseases that involve the oxidative stress, justifying the use of these compounds in folk medicine. 

## 4. General Aspects and the Role of Flavonoids in the Inflammation

Inflammation is a response to a cellular injury induced by physical stress, infectious agents, toxins, and other factors. Whereas an acute inflammatory reaction is important to the immune response and could culminate in a resolution of the injury, chronic inflammation can cause tissue destruction and are involved in the pathogenesis of autoimmune, neurodegenerative and respiratory diseases. The inflammatory response is part of the innate immune response. After an injury, the acute response mainly involves macrophage activation, which is a source of mediators such as histamine. Macrophages and other cells release pro-inflammatory cytokines such as tumor necrosis factor (TNF-α), interleukin-1 (IL-1) and IL-6, which trigger the inflammatory cascade by acting on end-organ receptors in response to injury [38,39]. These pro-inflammatory cytokines can induce endothelial cell leukocyte adhesion, the release of proteases the release of arachidonic acid metabolites, and the activation of the coagulation cascade [40]. Additionally, counter-regulatory and anti-inflammatory cytokines such as IL-10 provide a negative feedback system to counteract inflammatory cascade activation [41].

**Figure 3:**
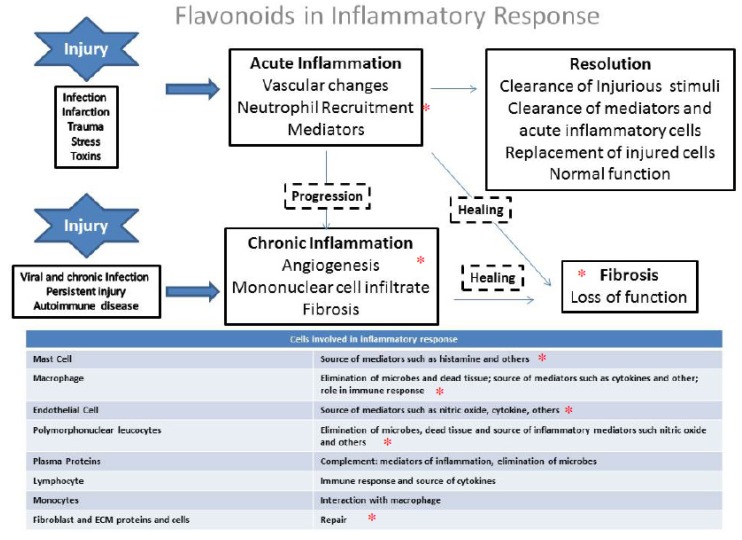
Inflammatory response and cells involved in inflammatory cascade. * shows the steps that provide evidence that flavonoids can act to counteract the inflammatory response.

Clinical and classical signals of inflammation are an increase in temperature, redness, swelling, and pain and tissue/organ dysfunction. The acute phase response is characterized by platelet adhesion, vasoconstriction of efferent vessels, afferent vascular dilation induced by cytokines release, activation of the complement and fibrinolytic systems, and the initiation of the leukocyte adhesion cascade. An increase in endothelial gaps allows extravasation of serum proteins (exudate) and leukocytes and culminates in tissue swelling and phagocytosis of foreign material with pus formation (Figure 3). 

Several inflammatory mediators are released during injury such as histamine, prostaglandins, thromboxanes and leukotrienes, as well as cytokines and nitric oxide [42,43]. Nitric oxide (NO) is produced when the amino acid L-arginine is converted in L-citrulline by nitric oxide synthase [44] using NG-hydroxyl-L-arginine as an intermediate [38]. Three NOS isoforms were described (two constitutive and one inducible): the neuronal or type I (nNOS), the inducible isoform or type II (iNOS), and the endothelial isoform or type 3 (eNOS) which is released primarily by endothelial cells of the injured tissue and exerts cytotoxic activity against microorganisms [38]. However, it has been previously demonstrated that in pathological situations, NO can be produced by the inducible isoform in high quantities and can affect lung inflammation [45,46]. Therefore, oxidative stress and nitrative stress are involved in the pathogenesis of inflammatory diseases [47]. 

The organism can resolve the inflammatory response *per se* (Figure 3). However, if the stimulus persists, a vicious cycle can be created, which involves the release of more and more cytokines, which in turn damages the tissue and induces the recruitment of more and more inflammatory cells, which causes the release of more cytokines and perpetuating the inflammation. Chronic inflammation is associated with various pathological situations including lung diseases, and is characterized by angiogenesis, mononuclear cell infiltrate and fibrosis and involves the release of several pro-inflammatory mediators. 

Prostaglandins (PGs) play a key role in the inflammatory response since an increased in PG biosynthesis is observed in inflamed tissue. The prostanoids PGs and thromboxane A2 (TXA2), are derived from the arachidonic acid (AA), a 20-carbon unsaturated fatty acid. Phospholipases release AAs in plasma membrane which are metabolized by the sequential actions of prostaglandin synthase or by cyclooxygenase (COX), which are found in two distinct isoforms known as COX-1 and COX-2 [48]. Commercially available drugs with anti-inflammatory effects are the most commonly used for inhibiting the COX enzyme to prevent the formation of inflammatory mediators such as prostaglandins and thromboxanes [49]. However, it is well known that these drugs trigger several adverse effects mainly because of the use of high doses and prolonged treatment. 

Flavonoids can suppress the expression of the COX gene through interactions with cell signaling pathways, such as the protein kinase C, NF-κB and tyrosine kinase pathways [50,51,52]. Previous studies have reported that flavonoids inhibit of arachidonic acid blocking the cyclooxygenase and 5-lipoxygenase pathways [53,54,55,56]. However, the mechanisms involved in this enzymatic inhibition are not fully elucidated. 

The anti-inflammatory effects of phenolic compounds are related to the ability in modulating the expression of pro-inflammatory genes, such as NOS, cyclooxygenase, lipoxygenase but also by acting throughout nuclear factor (NF-κB) signaling and mitogen-activated protein kinase [57,58] and activating the Nrf2/Keap1 pathway [59]. NF-κB has been reported as one of the most notable pro-inflammatory gene expression regulators and mediates several cytokines synthesis, such as TNF-α, IL-1β, IL-6, and IL-8 as well as COX-2 [60]. Previous studies have confirmed that flavonoids exert their anti-inflammatory effects by modulating the inflammatory cells, inhibiting the T lymphocyte proliferation, inhibiting pro-inflammatory cytokines (TNF-alpha and IL-1), or controlling enzymes derived from the arachidonic acid pathway such as NO formation [10,61,62,63,64,65].

Studies have shown that the flavonols such as quercetin (**18**) and kaempferol (**12**) exhibit significant anti-inflammatory actions which are believed to be through the inhibition of the phospholipase A2 (via arachidonic acid), lipoxygenase, cyclooxygenase and tromboxane enzymes and through the modulation of iNOS thereby inhibiting NO production [57,66]. Other flavonoids, such as myricetin (**17**), hesperetin (**6**) and naringenin (**2**), also exhibit inhibitory activity of the enzyme phospholipase A2 [64].

The inhibition of iNOS and NO production is also attributed to other flavonoids such as apigenin (**15**), luteolin (**19**), chrysin (**14**), kaempferol (**12**) and genistein (**42**) [57,64,65]. The flavonols morin (**22**) and myricetin (**17**) also have inhibitory actions on the enzyme lipoxygenase, the flavones chrysin (**14**), apigenin (**15**), and luteolin (**19**) and the flavonols morin (**22**), galangin (**23**) and rutin (**16**) inhibit the enzyme cyclooxygenase [64,66,67]. Genistein (**42**), quercetin (**18**), luteolin (**19**), apigenin (**15**) and rutin (**16**) are capable to inhibit pro-inflammatory cytokines such as TNF-α and IL-1 [61,62,64]. Luteolin (**19**), apigenin (**15**), myricetin (**17**) and quercetin (**18**) are also able to modulate lymphocytes and neutrophils [68]. Additionally, it is known that quercetin (**18**) inhibits the activity of cyclooxygenase and lipoxygenase metabolites [69,70].

An *in vitro* study shows that the flavonoids luteolin (**19**), apigenin (**15**) and fisetin (**24**) are potent inhibitors of the synthesis of IL-4 and IL-13 [71]. Scutellarein (**25**), 3-hydroxyflavone (**26**), kaempferol (**12**), quercetin (**18**), eriodictyol (**4**), fustin (**7**) and 7-hydroxyflavone (**27**) also act to inhibit the production of IL–4 with hydroxylation at positions C-3, C-5 and C-7 being necessary to observe the maximum inhibition. However, the precise mechanisms by which these flavonoids act in the control of cytokine synthesis remains to be better elucidated [71].

Additionally, the effect of the double bond in the flavonoid structure on the anti-inflammatory action, including the inhibition of lymphocyte proliferation, iNOS, COX and PLA-2, has been determined [10]. Structurally, the delocalization of electrons generated by the extension of π conjugation in the structure of flavonoids (ketonic carbonyl at C-4 and C-2/C-3 double bond at ring C) allows greater stability of intermediate radical species which is involved in inflammation process. Furthermore, the presence of a catechol group at the B ring is important because this group promotes enzymatic oxidation, inducing the formation of quinone/semiquinone electrophilic species that are capable of performnucleophilic addition. Thus, ligands act as electrophiles of macromolecules (e.g., proteins), resulting in reactions involving the formation of covalent bonds between the flavonoids and biomacromolecules. 

Furthermore, the flavanone hesperetin (**6**) displayed a lower inhibitory activity of the enzyme phospholipase A2 (PLA2) compared with the flavonols kaempferol (**12**), quercetin (**18**) and myricetin (**17**). This finding once again highlights the importance of the double bond at positions C-2/C-3 (ring C) for anti-inflammatory activity [10]. According to Lattig *et al*. [72] in their study of molecular modeling, the double bond between positions C-2/C-3 induces coplanarity between rings A and C, favoring the interaction of the flavonoid with the enzymatic site receptor.

Kim *et al*. [62] reported that the presence of hydroxyl groups at C-5 and C-7 (ring A) as well as at the C-3' and C-4' (ring B) positions is an important feature, while the glycosylation of these groups reduces the anti-inflammatory activity. Quercetin (**18**) and its glycoside derivative quercitrin (**13**) are an examples that reflect the importance of glycosylation. Both compounds inhibit the production of PGE2 and LTB4 in an induced pleurisy model in rats, but quercitrin (**13**) showed lower activity than their aglycone. A study by Rotelli *et al*. [73] also shows that glycosylation affects anti-inflammatory activity the classic model of carrageenan-induced edema in the mouse paw. The results indicate that aglycones (quercetin, **18** and hesperetin, **6**) gave positive results, while the glycosylated flavonoids showed no significant activity (rutin, **16** and hesperidin, **5**). 

Another comparative study between pairs of glycosides/aglycones [74] showed that the addition of sugar residues significantly reduces anti-inflammatory activity, suggesting that the lipophilicity and bioavailability of tested compounds becomes an important factor. Holmann *et al*. [75] suggested that quercetin glycoside derivatives are more readily absorbed than the aglycone forms. Nevertheless, these aspects are not fully elucidated because the role of glycosylation of flavonoids in facilitating absorption is questioned by the fact that catechin (**46**), which is not glycosylated in nature, is absorbed more efficiently [12]. Therefore, these observations suggest that glycosyl derivatives appear to be more active than those in aglycone forms [76].

**Figure 4:**
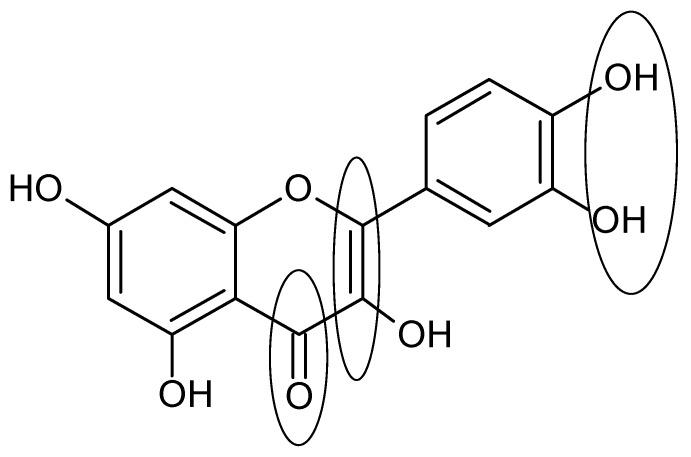
Structure of quercetin as example of important requisites (unsaturation in the C ring, number and position of hydroxyl groups at ring B, carbonyl group at C-4 and non-glycosylation) for anti-inflammatory activity of flavonoids.

In general, the currently structural requirements for anti-inflammatory activity of flavonoids (Figure 4) are the unsaturation in the C ring (Δ^2^), the number and position of hydroxyl groups (for example, the catechol group at ring B), the carbonyl group at C-4, and non-glycosylation of the molecule. However, compounds that do not have these structural features (e.g., the flavonoid aglycone kaempferol (**12**), also display an anti-inflammatory activity by affecting the enzymes of the inflammatory cascade [68].

## 5. The Role of Flavonoids in Lung Diseases

### 5.1. General Aspects

Generally, lung disease involves acute or chronic inflammation that culminates in a repair process of the lung and is highly associated with a decrement in lung function. Flavonoids have been frequently used in folk medicine and several studies have been conducted to prove that their effects are associated with anti-inflammatory and antioxidant properties, as described above.

Recently, Lim *et al.* [77], evaluating the pharmacological action of a total extract of *Morus alba* on airway inflammation, found a reduction in the total number of inflammatory cells and a reduction in TNF-α, IL-6 and NO production by lung macrophages. The crude extract also inhibited epithelial hyperplasia and alveolar space destruction in a model of airway inflammation. Therefore, treatment with antioxidant and anti-inflammatory drugs may be beneficial in inhibiting or reducing the progression of pulmonary disease in patients with COPD, lung cancer, acute respiratory distress syndrome (ARDS) and asthma. Additionally, some reports suggest that nutrition, with an emphasis on a high consumption of fruits and vegetables, rich in flavonoids and other antioxidant compounds, can be an important element of primary prevention of cancer [78].

### 5.2. Chronic Obstructive Pulmonary Disease (COPD)

The incidence and severity of chronic obstructive pulmonary disease (COPD) is growing affecting between 100 and 150 million people worldwide, is related to a significant rate of mortality [79] occupying the fourth leading cause of death worldwide. COPD is characterized by persistent airflow limitation that is usually progressive and is associated with an enhanced chronic inflammatory response to noxious particles or gases in the airways and the lung [79]. The inhalation of cigarette smoke and other environmental pollutants stimulates the alveolar macrophages in the lung epithelial cells to generate reactive oxygen species (ROS) and reactive nitrogen species (RNS) in excess causing an imbalance in the system [80,81]. The inflammatory cells recruited by oxidative stress as well as the local epithelial cells produce matrix metalloproteinases (MMPs) in the lungs, which in excess can degrade the alveolar walls and lead to an increased air space and the development of emphysema [82]. Although the effort to understand the cellular and molecular mechanisms involved in the pathogenesis of COPD, these aspects are not well elucidated until now; it is believed that oxidative stress, chronic inflammation and an imbalance of proteases and antiproteases are involved in the development and evolution of the emphysema [80,81,83]. 

Several flavonoids such as catechins, flavonols and flavones are positively associated with force expiratory volume in one second (FEV1) increment and inversely associated with symptoms such as chronic cough, shortness of breath (not chronic) and phlegm, suggesting a beneficial effect of ingestion of catechins in patients with COPD. Studies have shown beneficial effects of flavonoids, such as quercetin (**18**), on COPD and in the prevention of the progression of pulmonary emphysema in animal models [84].

Poly (ADP-ribose) polymerase (PARP)-1 is a nuclear enzyme involved in the pro-inflammatory signal transduction pathway of chronic obstructive pulmonary disease (COPD). In addition, oxidative stress is the main pathological mechanism in COPD. Therefore, an experiment evaluating several types of anti-inflammatory flavonoids—fisetin (**24**), morin (**22**), flavone (**29**) and tricetin (**34**)—was performed to examine the involvement of molecular signaling. Blood samples from patients diagnosed with COPD were pre-incubated with the flavonoids and then exposed to LPS: the concentration of TNF-α, IL-6, IL-8 and IL-10 were then measured. Tricetin (**34**) and fisetin (**24**) were the most efficient treatment for attenuating LPS-induced increases of TNF-α. These flavonoids were also able to attenuate cytokine release from leukocytes, thus making the PARP-1-inhibiting compounds potential nutraceutical agents for patients with the COPD [85].

Quercetin (**18**), another widely studied flavonoid [86,87,88,89,90], has been assessed for the release of elastase that is the major product secreted by neutrophils, which are cells associated with the innate immune system. When stimulated, neutrophils release elastase, which is mainly responsible for degrading foreign proteins during phagocytosis. Though imbalanced, the nonspecific enzyme release can damage cell membranes and extracellular space proteins such as elastin, fibronectin and eoglycans, causing several inflammatory diseases such as respiratory distress syndrome, pulmonary emphysema and ARDS [91]. In an evaluation of immunomodulatory activity evaluation, along myricetin (**17**), kaempferol (**6**) and quercetin (**18**) proved to be the most potent inhibitors of elastase release, suggesting, a therapeutic use for neutrophil-dependent inflammatory diseases [92]. 

Furthermore, it is well-accepted that quercetin (**18**) has a protective effect on cells, the cytoprotective mechanism of which has been extensively investigated. This flavonoid upregulates endogenous defensive pathways and activates specific transcriptional factors. A study performed with the LA-4 cell line substantiated the findings that quercetin (**18**) increases in the levels of heme oxygenase (HO)-1 expression, protecting cells from the H_2_O_2_ apoptotic effects, such as hypodiploid cells, the activation of caspase 3 enzyme activity and lactate dehydrogenase release. Therefore, quercetin (**18**) successfully attenuates oxidative epithelial cell injury in lung inflammation [88].

The modulatory effect of quercetin (**18**) on protein expression has also been investigated for cytoprotection in airway inflammatory diseases. In human epithelial cells (HBE16), the effect of this flavonoid on mucin 5AC (MU5AC) expression induced by human neutrophil elastase was evaluated [37]. Quercetin (**18**) reduced both gene transcription and protein expression of MUC5AC in a dose-dependent manner and also markedly decreased mRNA and protein expression of epidermal growth factor (EGFR). Phosphorylated ERK 1/2 which was significantly increased with HNE treatment, was also decreased by quercetin (**18**), as was pPKC HNE-induced protein expression. Altogether, quercetin (**18**) most likely intervenes in the PKC/EGFR/ERK signal transduction pathways to inhibit HN-induced MUC5AC expression in human airway epithelial cells, making this flavonoid a potential treatment for counteracts mucus hypersecretion in chronic inflammatory airway disease [89].

Moreover, quercetin (**18**) exhibits anti-inflammatory effects in addition to the inhibition of protein tyrosine and serine/threonine kinases [93]. This flavonoid has been shown to reduce inflammation and lung expression of MMP9 and MMP12, by increasing type III deacetylase protein Sirt-1 expression, a down-regulator of MMP both *in vivo* and *in vitro* [94]. Furthermore, this flavonoid was effective in improving the elasticity of the lung and prevented the progressive loss of elastic recoil and subsequent progression of emphysema, but did not stimulate the regeneration of damaged alveoli. 

The flavonoid liquiritin apioside (**9**) was evaluated in the human type II alveolar epithelial cell line (A 549) for protective effects against cigarette smoke-mediated oxidative stress. Treatment with this flavonoid resulted in a concentration-dependent prevention of cigarette smoke extract (CSE)-induced cytotoxicity, an increase of TGF-β and TNF-α mRNA expression, depletion of glutathione (GSH) and cell apoptosis. In treated ICR mice, this flavonoid displayed dose-dependent inhibition of pulmonary neutrophils and macrophage inflammation and inhibited the presence of mucus-containing globet cells in the proximal airways. Moreover, TGF-β and TNF-α release and myeloperoxidase activity induced by chronic smoke was attenuated, whereas the superoxide dismutase activity was improved [95]. These authors suggested that the liquiritin apiodise (**45**) protects lung epithelial cell injury induced by cigarette smoke by inhibiting TGF-β and TNF-α and increasing the GSH levels, suggesting a potential role as an agent against epithelial damage in COPD.

The flavonoid baicalin (**33**) was also tested in COPD, a condition considered insensitive to corticosteroids therapies. This flavonoid was able to reduce inflammation by decreasing inflammatory cells and the production of TNF-α, IL-8 and MMP-9 in BalB/C mice submitted to cigarette smoke exposures, results that were not achieved by the positive control group treated with the synthetic corticosteroid dexamethasone.

### 5.3. Acute Respiratory Distress Syndrome (ARDS)

ARDS is a common disease in the Intensive Care Unit (ICU), characterized by increased infiltration of inflammatory cells (mainly neutrophils), by the production of inflammatory mediators, such as TNF-α and of reactive oxygen species (ROS), tissue edema and extracellular matrix remodeling [96]. ARDS is mainly associated with lung infection but also has extra-pulmonary causes. Although the control of lung ventilation as a protection strategy has considerably reduced mortality, no specific treatment is available; the number of young people who died because of ARDS is high and the treatment only considered the cause. Some evidence suggests that substances of the flavonoid class may have a modulatory effect in ARDS, but most of the studies are in experimental models [97,98]. 

Models of lipopolysaccharide-induced lung inflammation are also used to study the anti-inflammatory effects of flavonoids because LPS is present in the membrane of gram-negative bacteria and LPS is considered one of the main risk factors for ARDS [97]. In a study evaluating the protective activity of the flavonoid luteolin (**19**) in animals that received LPS to mimic ARDS, authors showed an important reduction of lung hemorrhage, neutrophilic inflammation as well as interstitial edema in distal lung parenchyma and alveolar spaces [99]. The reduction of catalase activity, superoxide dismutase activity, oxidative damage and lipid peroxidation has also been achieved in lung tissues after luteolin (**19**) pretreatment and LPS challenge. Moreover, the authors also found a reduction in TNF-α, KC and ICAM-1 content in BALF cells induced by luteolin (**19**). Regarding the mechanism of action, this flavonoid reduced LPS-induced activation of the MAPK and NFκB pathways [99]. Other works have also studied flavonoids for their beneficial effects in LPS-induced ARDS [97].

The protective effect of kaempferol (**12**), as well as its mechanism of action, has been evaluated in LPS-induced ARDS. This flavonoid, administered to BALB/c mice intragastrically attenuated pulmonary edema, markedly decreased lung wet-to-dry ratio, and decreased protein concentration and inflammatory cells in BALF. Through histological studies was possible to find that this flavonol substantially attenuated alveolar wall thickness and hemorrhage as well as inflammatory cells infiltration in lung parenchyma induced by LPS; however, superoxide dismutase activity increased. It was suggested that the mechanism of kaempferol’s (**12**) benefic effect in LPS-induced ARDS was via the controlling of the MAPK and NF-κB signaling pathways, with inhibition of tissue oxidative stress and pulmonary inflammatory response most likely involved as well [100]. Acting on toll-like receptor 4 (TLR4), a < 20 μM concentration of nontoxic kaempferol (**12**) attenuated the LPS-induced IL-8 release and inhibited eotaxin-1 induction as well [101]

The flavanone pinocembrin (**10**) has been assessed for its therapeutic effects in LPS-induced ARDS. In a murine model, the pulmonary edema, histological severity, neutrophilic, lymphocytic and macrophagic infiltrations were attenuated. Pretreatment using pinocembrin (**10**) also decreased the concentrations of TNF-α, IL-1 β, IL-6 and increased IL-10 levels. Parallel *in vitro* experiments suggested that the mitigation of LPS-induced ARDS by this flavonoid is a result of the suppression of Iκ α, JNK and p38MAPK activation [102].

Also assessed in LPS-induced lung inflammation, the flavonoid oroxylin-A (**32**) has been tested for its beneficial anti-inflammatory effects in *in vivo* experiments. Isolated from *Scutellaria baicalensis* [103], this compound mitigated reduced inflammation, elevated levels of TNF-α and NO in the plasma, alveolar edema and thickened alveolar septa through a post-treatment administration. Altogether, oroxylin-A (**32**) proved to be a potential drug for ameliorating lung inflammation induced by LPS [98].

Additionally, the anti-inflammatory and antioxidant protective properties of quercetin (**18**) were assayed against pneumotoxic substances that cause lung injury. Paraquat (PQ: 1,1'-dimethyl-4-4'-bipyridinium) is thought to induce pulmonary damage via reduction in redox cyclic, creating PQ cation radicals that result in oxidation of NADPH to NADP^+^ in association to superoxide anion and other oxygen free radicals [86]. An *in vivo* experiment the authors showed that exposure to PQ induced an elevated pulmonary malondialdehyde (MDA) levels, heme oxygenase-1 (HO-1) expression and total oxyradical scavenging capacity reduction. These alterations were inhibited by post-treatment with quercetin (**18**). This flavonoid treatment also significantly inhibited the NO, MDA and 4-hydroxyproline (4-HP) elevation and pulmonary glutathione (GSH) reduction. Therefore, the antioxidant activity of quercetin (**18**) was most likely responsible for inhibiting the development of PQ-induced pulmonary injury [86].

Naringin (**1**), a flavanone derivative, was also tested for in paraquat-induced ARDS and pulmonary fibrosis. Daily treatment with naringin (**1**) highly increased survival rates in PQ exposed mice and improved, in a dose-dependent manner, increased leukocyte infiltration and the overexpression of TNF-α and TGF-β1 resulting from PQ challenge. Treatment with narigin (**1**) also reduced the PQ-induced upregulation of MMP-9 and its inhibitor TIMP-1, pulmonary malonaldehyde and hydroxyproline as well as pulmonary fibrosis. These features were associated to increased superoxide dismutase, glutathione peroxidase (GSH-Px) and heme-oxygenase (HO-1) activities. Therefore, naringin (**1**) is a flavonoid that exhibits effective protection against PQ-induced lung injury [104].

### 5.4. Asthma

Asthma, a chronic airway inflammatory disorder, is a serious public health problem all over the world, [105]. Recurrent episodes of wheezing, chest tightness, breathlessness and coughing are present in patients with asthma which are associated to airway hyper-responsiveness and chronic airway inflammation. These episodes are usually associated with extensive, airflow obstruction within the lung that is frequently reversible either spontaneously or with treatment. There is an important role of cytokines in asthma which orchestrate the inflammatory response and determine the crisis severity. Several cytokines are involved in asthmatic physiopathology including Th2 classifical cytokines such as IL-5, IL-4 and IL-13, which are required for eosinophil differentiation and survival, for TH2 cell differentiation and for IgE formation.respectivelly. In addition, IL-1β and TNF-α, are also important and acts amplifying the inflammatory response, and GM-CSF, which prolongs airways eosinophil survival. Mast cells release mediators such as histamine which contributes to airway smooth muscle constriction and inflammation [106].

Several studies have shown that food habits can influence to the development of allergic diseases. Devereux *et al.* [107] showed that low consumption of foods containing antioxidants such as fruits and vegetables may be associated with an increase in asthma and atopic diseases, while the ingestion of antioxidants and lipids during pregnancy and childhood can reduce the appearance of allergic diseases. A case-controlled, population-based study reported that the consumption of apples and red wine was negatively associated with the asthma prevalence or severity, possibly attributed to the beneficial effect of flavonoids [108]. In addition, these authors also suggested that an increase in the flavonoid ingestion decreases the incidence of asthma in the population. 

The anti-allergic capacity of flavonoids such as apigenin (**15**), luteolin (**19**), 3,6-dihydroxy-flavone (**28**), fisetin (**24**), kaempferol (**12**), quercetin (**18**), and myricetin (**17**) can be associated with a decrease in histamine release [35,109,110] through the inhibition of the chemical mediators and cytokine production by mast cells and with the suppression of the cysteinyl leukotriene synthesis through the inhibition of phospholipase (PLA2) and/or 5-lipoxygenase (5LO) [111]. However, the molecular mechanism involved is not totally clear. Townsend *et al*. [112] using tracheal rings *in vitro,* found an airway smooth muscle relaxation induced by quercetin (**18**) which also potentiates the relaxation effects of β-agonist via dual phosphodiesterase inhibition of prostaglandins. Additionally, an *in vitro* study evaluating the effects of kaempferol (**12**) in lung inflammation was conducted using the human airway epithelial BEAS-2B cell line found a reduction in inflammation. In addition, in an *in vitro* experiment, BALB/c OVA-sensitized mice were used to demonstrate the demoting effect of this flavonoid on asthmatic inflammation. Through oral administration, kaempferol (**12**) alleviated airway inflammation by modulating tyrosine kinase 2-STAT1/3 and IL-8, both for airway epithelium and in the asthmatic [101]. 

The flavonoids fisetin (**24**) and morin (**22**) and kaempferol (**12**) have also been tested in asthma models both on the early response and in the late response. In ovalbumin sensitized guinea pigs, all three flavonols reduced the recruitment of total leukocytes. Histamine content, phospholipase A2 and EPO (eosinophil peroxidase) activity were also significantly inhibited by the flavonols. Kaempferol (**12**) treatment resulted in the greatest anti-asthmatic effect, and fisetin (**24**) treatment produced the highest inhibition of Raw (airway resistance). Such data suggests that the lower the molecular weight, the greater the beneficial effects on asthma of these compounds [113].

Other flavonoids such as quercetagetin (35), kaempferol-3-O-galactoside (36) and scutellarein (25) are phenolic compounds that acts inhibiting the PLA2 [56]. Cirsiliol (37) is able to inhibit by 99%, the activity of leukotrienes. In addition, quercetin (18), luteolin (19) and baicalein (30) were able to inhibit the secretion of granulocyte-macrophage colony-stimulating factor (GM-CSF) in cultures of human mast cells in response to the FcεRI44 crosslinker [114] besides counteracts the release of histamine, leukotriene and prostaglandin D2. These compounds also affect the release of IgE by bone marrow-derived cultures murine mast cell [115]. Studies performed in an *in vivo* model of allergic airway inflammation have shown that quercetin (18) acts as an inhibitor of TNF-α, thus reducing airway responsiveness [116], and also inhibits eosinophilic inflammation in asthmatic animals [117]. 

Furthermore, flavonoids such as luteolin (**19**), apigenin (**15**) and fisetin (**24**) have been shown to significantly decrease the synthesis of IL-4, IL-13 and TNF-α synthesis [71,118,119,120], while isoquercitrin (**38**) and quercetin (**18**) reduce the recruitment of eosinophils and the formation of leukotrienes in an experimental asthma model in mice [117]. Moreover, luteolin (**19**), apigenin (**15**) and fisetin (**24**) suppress the mRNA expression of IL-4, IL-13 and CD40 ligand as well as their respective protein activities, indicating that these flavonoids could act as natural inhibitors of IgE [71].

A study involving five flavonoids isolated from *Glycyrrhiza uralensis* (Gan-Cao), a Chinese plant, investigated the ability of the compounds to inhibit human fetal lung fibroblast (HFL-1) secretion of eotaxin-1 [118]. Liquiritigenin (**8**), isoliquiritigenin (**45**) and 7,4-dihydroxyflavone (**31**) proved to be effective in demonstrating anti-inflammatory activity [121]. Other flavonoids derived from the same species have been investigated for their protective effect against respiratory diseases.

Genistein (**42**), a phytoestrogen belonging to the isoflavone class, has been studied in different conditions involving airway inflammation. Considered a broad-spectrum protein tyrosine kinase inhibitor, genistein (**42**) (15 mg/kg administered intraperitoneally to sensitized male Hartley guinea pigs) inhibited ovalbumin-induced acute bronchoconstriction and airway hyperresponsiveness to methacholine, eosinophilic inflammation both in BALF and in airways and eosinophil peroxidase activity in cell-free assays. The authors suggested that the inhibition of the tyrosine signaling cascade by via genistein (**42**) stimulus may present a therapeutic possibility for allergic airway diseases [122]. 

Soy isoflavones containing high levels of genistein (**42**) (over 40%) and other derivatives, such as daidzein (**44**) and daidzin (**43**), were used in a murine model of allergic asthma. The oral administration of increase doses of soy isoflavones significantly attenuated OVA-induced AHR in response to intravenous methacholine and also reduced the eosinophils count in lung. Daily administration also markedly suppressed, in a dose dependent manner, the eotaxin, IL-5, IL-4 and matrix metalloproteinase-9 (MMP-9) mRNAs, augment the expression of interferon (IFN-γ) mRNA and the expression of tissue inhibitor of metalloproteinase-1 (TIMP-1). Moreover, these compounds also inhibit airway mucus production and extracellular matrix remodeling in lung tissues which can be observed through histological studies [123]. 

Finally, due to the anti-inflammatory potential and antioxidant activity of sakuranetin (**11**) [124], our research group recently tested the effects of this flavonoid in models of lung disease [58]. We showed that sakuranetin (**11**) has a great potential to treat asthma since it reduced eosinophilic inflammation and reversed lung remodeling in mice. In addition, we demonstrated that these effects could be attributed to a reduction in Th2 cytokines (but not Th1 cytokines), oxidative stress and the control of NF-κB activity. All of these effects contribute to reduce the airway hyperresponsiveness observed in this animal model. Preliminary studies also suggest that sakuranetin (**11**) reduces lung emphysema as well as neutrophilic and macrophage inflammation [125], suggesting a potential use of this compound in treat emphysema. Table 1 lists the main flavonoids that have displayed activity in lung inflammation and diseases.

**Table 1:** Active flavonoids in lung inflammation and diseases.

Flavonoid	Lung Effect	Reference
Naringin (1)	Anti-ARDS	[104]
Kaempferol (12)	Anti-allergic	[35,109,110]
	Anti-ARDS	[100]
	Anti-asthmathic	[101]
Apigenin (15)	Anti-allergic	[35,109,110]
	Anti-asthmathic	[71,120]
Genistein (42)	Bronchoconstrictor	[122]
Daidzin (43)	Bronchoconstrictor	[123]
Daidzein (44)	Bronchoconstrictor	[123]
Myricetin (17)	Anti-allergic	[35,109,110]
Quercetin (18)	Preventing pulmonar emphysema	[84]
	Anti-allergic	[35,109,110]
	Anti-ARDS	[91]
	Anti-asthmathic	[89,114,117]
Luteolin (19)	Anti-allergic	[35,109,110]
	Anti-asthmathic	[71,114,120]
	Anti-COPD	[97,99]
Morin (22)	Anti-asthmathic	[113]
	Anti-COPD	[85]
Fisetin (24)	Anti-allergic	[35,109,110]
	Anti-asthmathic	[113]
	Anti-COPD	[85]
Scutellarein (25)	Anti-asthmathic	[115]
3,6-Dihydroxyflavone (28)	Anti-allergic	[35,109,110]
Flavone (29)	Anti-COPD	[85]
Tricetin (34)	Anti-COPD	[85]
Quercetagetin (35)	Anti-asthmathic	[56]
Kaempferol-3-O-galactoside (36)	Anti-asthmathic	[56]
Cirsiliol (37)	Anti-asthmathic	[115]
Baicalein (30)	Anti-asthmathic	[115]
Isoquercitrin (38)	Anti-asthmathic	[71,117]
Liquiritigenin (8)	Anti-COPD	[121]
Isoliquiritigenin (45)	Anti-COPD	[121]
7,4’-Dihydroxyflavone (31)	Anti-COPD	[121]
Liquiritin apioside (9)	Anti-COPD	[95]
Pinocembrin (10)	Anti-ARDS	[102]
Oroxylin-A (32)	Act decreasing lung inflamattion	[98]
Baicalin (33)	Anti-COPD	[126]
Sakuranetin (11)	Anti-asthmathic and COPD	[58,125]

## 6. Clinical Perspectives of Flavonoid Application in Lung Disease

Although several studies have shown that different types of flavonoids are beneficial in controlling respiratory diseases, few have been performed in humans. As described in this review, the majority of the studies in humans are with asthmatic patients; there were no studies evaluating emphysematous patients and only a few evaluating ARDS patients. Notwithstanding the small number of reports, the results are promising because most of the studies revealed positive results of phytotherapy on lung diseases. Kalhan *et al*. [127] showed that the asthmatic patients receiving a soy isoflavone supplement for four weeks exhibited a reduction in exhaled NO and *ex vivo* production of leukotrienes by eosinophils obtained from the blood of these patients. In addition, Smith [128] demonstrated that this flavonoid is associated with improved lung function in more than 1,000 patients studied. Most studies suggest that the mechanism of action of flavonoids is through the inhibition of NF-kB. Despite of these results, additional studies of the specific effects of flavonoids on molecular mechanisms in lung diseases and studies evaluating toxic adverse effects especially in humans are need.

## 7. Conclusions

In this review, we report that the main effect of flavonoids in lung disease could be attributed to an antioxidant and anti-inflammatory effect. Inflammation is involved in all lung diseases, and its inhibition could ameliorate lung function as well as avoid lung remodeling. Several flavonoids have been tested in experimental models and were found to be beneficial, mainly by inhibiting cytokines associated with the down-regulation of several transcription factors. Generally, the key structural features of active flavonoids are unsaturation in the C ring (∆^2^), the number and position of hydroxyl groups at the A and B rings, the carbonyl group at C-4 at ring C and frequently, non-glycosylation of the molecule. Taking these features into consideration, one may infer that natural products such as flavonoids may possibly be useful as prototypes in the development of novel substances for the treatment of several lung illnesses. However, further investigation is needed to evaluate the precise mechanisms of action and to determine if these results can be applied to diseases in humans. 

## References

[1] Bravo L. (1998). Polyphenols: Chemistry, dietary sources, metabolism, and nutritional significance. Nutr. Rev..

[2] Caltagirone S., Ranelletti F.O., Rinelli A., Maggiano N., Colasante A., Musiani P., Aiello F.B., Piantelli M. (1997). Interaction with type II estrogen binding sites and antiproliferative activity of tamoxifen and quercetin in human non-small-cell lung cancer. Am. J. Respir. Cell Mol. Biol..

[3] Ranelletti F.O., Ricci R., Larocca L.M., Maggiano N., Capelli A., Scambia G., Benedetti-Panici P., Mancuso S., Rumi C., Piantelli M. (1992). Growth-inhibitory effect of quercetin and presence of type-II estrogen-binding sites in human colon-cancer cell lines and primary colorectal tumors. Int. J. Cancer.

[4] Yoshida M., Sakai T., Hosokawa N., Marui N., Matsumoto K., Fujioka A., Nishino H., Aoike A. (1990). The effect of quercetin on cell cycle progression and growth of human gastric cancer cells. FEBS Lett..

[5] Lee K.H., Yoo C.G. (2013). Simultaneous inactivation of GSK-3beta suppresses quercetin-induced apoptosis by inhibiting the JNK pathway. Am. J. Physiol. Lung Cell Mol. Physiol..

[6] Croft K.D. (1998). The chemistry and biological effects of flavonoids and phenolic acids. Ann. N. Y. Acad. Sci..

[7] Graf B.A., Milbury P.E., Blumberg J.B. (2005). Flavonols, flavones, flavanones, and human health: Epidemiological evidence. J. Med. Food.

[8] Veitch N.C., Grayer R.J. (2008). Flavonoids and their glycosides, including anthocyanins. Nat. Prod. Rep..

[9] Boots A.W., Haenen G.R., Bast A. (2008). Health effects of quercetin: From antioxidant to nutraceutical. Eur. J. Pharmacol..

[10] Middleton E., Kandaswami C., Theoharides T.C. (2000). The effects of plant flavonoids on mammalian cells: Implications for inflammation, heart disease, and cancer. Pharmacol. Rev..

[11] Williams C.A., Grayer R.J. (2004). Anthocyanins and other flavonoids. Nat. Prod. Rep..

[12] Nijveldt R.J., van Nood E., van Hoorn D.E., Boelens P.G., van Norren K., van Leeuwen P.A. (2001). Flavonoids: A review of probable mechanisms of action and potential applications. Am. J. Clin. Nutr..

[13] Beecher G.R. (2003). Overview of dietary flavonoids: Nomenclature, occurrence and intake. J. Nutr..

[14] Das D.K. (1994). Naturally occurring flavonoids: Structure, chemistry, and high-performance liquid chromatography methods for separation and characterization. Methods Enzymol..

[15] Jorgensen R. (1993). The origin of land plants: A union of alga and fungus advanced by flavonoids?. Biosystems.

[16] Calixto J.O.B. (2003). Biodiversidade como fonte de medicamentos. Ciênc. Cult..

[17] Newman D.J., Cragg G.M. (2007). Natural products as sources of new drugs over the last 25 years. J. Nat. Prod..

[18] Soobrattee M.A., Neergheen V.S., Luximon-Ramma A., Aruoma O.I., Bahorun T. (2005). Phenolics as potential antioxidant therapeutic agents: Mechanism and actions. Mutat. Res..

[19] Lien E.J., Ren S., Bui H.H., Wang R. (1999). Quantitative structure-activity relationship analysis of phenolic antioxidants. Free Radic. Biol. Med..

[20] Bors W., Heller W., Michel C., Saran M. (1990). Flavonoids as antioxidants: Determination of radical-scavenging efficiencies. Methods Enzymol..

[21] Silva M.M., Santos M.R., Caroco G., Rocha R., Justino G., Mira L. (2002). Structure-antioxidant activity relationships of flavonoids: A re-examination. Free Radic. Res..

[22] Cao G., Sofic E., Prior R.L. (1997). Antioxidant and prooxidant behavior of flavonoids: Structure-activity relationships. Free Radic. Biol. Med..

[23] Rice-Evans C.A., Miller N.J., Paganga G. (1996). Structure-antioxidant activity relationships of flavonoids and phenolic acids. Free Radic. Biol. Med..

[24] Wolfe K.L., Liu R.H. (2008). Structure-activity relationships of flavonoids in the cellular antioxidant activity assay. J. Agric. Food Chem..

[25] Noroozi M., Angerson W.J., Lean M.E. (1998). Effects of flavonoids and vitamin C on oxidative DNA damage to human lymphocytes. Am. J. Clin. Nutr..

[26] Ioku K., Tsushida T., Takei Y., Nakatani N., Terao J. (1995). Antioxidative activity of quercetin and quercetin monoglucosides in solution and phospholipid bilayers. Biochim. Biophys. Acta.

[27] Mao T.K., Powell J., van de Water J., Keen C.L., Schmitz H.H., Hammerstone J.F., Gershwin M.E. (2000). The effect of cocoa procyanidins on the transcription and secretion of interleukin 1 beta in peripheral blood mononuclear cells. Life Sci..

[28] Burda S., Oleszek W. (2001). Antioxidant and antiradical activities of flavonoids. J. Agric. Food Chem..

[29] Huk I., Brovkovych V., Nanobash Vili J., Weigel G., Neumayer C., Partyka L., Patton S., Malinski T. (1998). Bioflavonoid quercetin scavenges superoxide and increases nitric oxide concentration in ischaemia-reperfusion injury: An experimental study. Br. J. Surg..

[30] Shutenko Z., Henry Y., Pinard E., Seylaz J., Potier P., Berthet F., Girard P., Sercombe R. (1999). Influence of the antioxidant quercetin *in vivo* on the level of nitric oxide determined by electron paramagnetic resonance in rat brain during global ischemia and reperfusion. Biochem. Pharmacol..

[31] Van Acker S.A., Tromp M.N., Haenen G.R., van der Vijgh W.J., Bast A. (1995). Flavonoids as scavengers of nitric oxide radical. Biochem. Biophys. Res. Commun..

[32] Cos P., Ying L., Calomme M., Hu J.P., Cimanga K., van Poel B., Pieters L., Vlietinck A.J., Vanden Berghe D. (1998). Structure-activity relationship and classification of flavonoids as inhibitors of xanthine oxidase and superoxide scavengers. J. Nat. Prod..

[33] Ferrali M., Signorini C., Caciotti B., Sugherini L., Ciccoli L., Giachetti D., Comporti M. (1997). Protection against oxidative damage of erythrocyte membrane by the flavonoid quercetin and its relation to iron chelating activity. FEBS Lett..

[34] Friesenecker B., Tsai A.G., Intaglietta M. (1995). Cellular basis of inflammation, edema and the activity of Daflon 500 mg. Int. J. Microcirc. Clin. Exp..

[35] Middleton E., Kandaswami C. (1992). Effects of flavonoids on immune and inflammatory cell functions. Biochem. Pharmacol..

[36] Zhu B.T., Ezell E.L., Liehr J.G. (1994). Catechol-*O*-methyltransferase-catalyzed rapid *O*-methylation of mutagenic flavonoids. Metabolic inactivation as a possible reason for their lack of carcinogenicity *in vivo*. J. Biol. Chem..

[37] Fiander H., Schneider H. (2000). Dietary ortho phenols that induce glutathione S-transferase and increase the resistance of cells to hydrogen peroxide are potential cancer chemopreventives that act by two mechanisms: The alleviation of oxidative stress and the detoxification of mutagenic xenobiotics. Cancer Lett..

[38] Ricciardolo F.L., Sterk P.J., Gaston B., Folkerts G. (2004). Nitric oxide in health and disease of the respiratory system. Physiol. Rev..

[39] Matot I., Sprung C.L. (2001). Definition of sepsis. Intensive Care Med..

[40] Wheeler A.P., Bernard G.R. (1999). Treating patients with severe sepsis. N Engl. J. Med..

[41] Pajkrt D., van der Poll T., Levi M., Cutler D.L., Affrime M.B., van den Ende A., ten Cate J.W., van Deventer S.J. (1997). Interleukin-10 inhibits activation of coagulation and fibrinolysis during human endotoxemia. Blood.

[42] Hesslinger C., Strub A., Boer R., Ulrich W.R., Lehner M.D., Braun C. (2009). Inhibition of inducible nitric oxide synthase in respiratory diseases. Biochem. Soc. Trans..

[43] Holgate S.T. (1997). Asthma: A dynamic disease of inflammation and repair. Ciba Found. Symp..

[44] Hantos Z., Adamicza A., Janosi T.Z., Szabari M.V., Tolnai J., Suki B. (2008). Lung volumes and respiratory mechanics in elastase-induced emphysema in mice. J. Appl. Physiol..

[45] Prado C.M., Leick-Maldonado E.A., Yano L., Leme A.S., Capelozzi V.L., Martins M.A., Tiberio I.F. (2006). Effects of nitric oxide synthases in chronic allergic airway inflammation and remodeling. Am. J. Respir. Cell Mol. Biol..

[46] Prado C.M., Leick-Maldonado E.A., Kasahara D.I., Capelozzi V.L., Martins M.A., Tiberio I.F. (2005). Effects of acute and chronic nitric oxide inhibition in an experimental model of chronic pulmonary allergic inflammation in guinea pigs. Am. J. Physiol. Lung Cell Mol. Physiol..

[47] Ricciardolo F.L., di Stefano A., Sabatini F., Folkerts G. (2006). Reactive nitrogen species in the respiratory tract. Eur. J. Pharmacol..

[48] Smith W.L., DeWitt D.L., Garavito R.M. (2000). Cyclooxygenases: Structural, cellular, and molecular biology. Annu. Rev. Biochem..

[49] Rang H.P., Dale M.M., Ritter J.M., Flower R.J. (2007). Farmacologia.

[50] Lin S.Y., Tsai S.J., Wang L.H., Wu M.F., Lee H. (2002). Protection by quercetin against cooking oil fumes-induced DNA damage in human lung adenocarcinoma CL-3 cells: Role of COX-2. Nutr. Cancer.

[51] Bastianetto S., Zheng W.H., Quirion R. (2000). Neuroprotective abilities of resveratrol and other red wine constituents against nitric oxide-related toxicity in cultured hippocampal neurons. Br. J. Pharmacol..

[52] Lee S.C., Kuan C.Y., Yang C.C., Yang S.D. (1998). Bioflavonoids commonly and potently induce tyrosine dephosphorylation/inactivation of oncogenic proline-directed protein kinase FA in human prostate carcinoma cells. Anticancer Res..

[53] Ferrandiz M.L., Alcaraz M.J. (1991). Anti-inflammatory activity and inhibition of arachidonic acid metabolism by flavonoids. Agents Actions.

[54] Ferrandiz M.L., Nair A.G., Alcaraz M.J. (1990). Inhibition of sheep platelet arachidonate metabolism by flavonoids from Spanish and Indian medicinal herbs. Pharmazie.

[55] Laughton M.J., Evans P.J., Moroney M.A., Hoult J.R., Halliwell B. (1991). Inhibition of mammalian 5-lipoxygenase and cyclo-oxygenase by flavonoids and phenolic dietary additives. Relationship to antioxidant activity and to iron ion-reducing ability. Biochem. Pharmacol..

[56] Yoshimoto T., Furukawa M., Yamamoto S., Horie T., Watanabe-Kohno S. (1983). Flavonoids: Potent inhibitors of arachidonate 5-lipoxygenase. Biochem. Biophys. Res. Commun..

[57] Santangelo C., Vari R., Scazzocchio B., di Benedetto R., Filesi C., Masella R. (2007). Polyphenols, intracellular signalling and inflammation. Ann. Ist. Super Sanita.

[58] Toledo A.C., Sakoda C.P., Perini A., Pinheiro N.M., Magalhaes R.M., Grecco S., Tiberio I.F., Camara N.O., Martins M.A., Lago J.H. (2013). Flavonone treatment reverses airway inflammation and remodelling in an asthma murine model. Br. J. Pharmacol..

[59] Jiang J., Mo Z.C., Yin K., Zhao G.J., Lv Y.C., Ouyang X.P., Jiang Z.S., Fu Y., Tang C.K. (2012). Epigallocatechin-3-gallate prevents TNF-alpha-induced NF-kappaB activation thereby upregulating ABCA1 via the Nrf2/Keap1 pathway in macrophage foam cells. Int. J. Mol. Med..

[60] Tak P.P., Firestein G.S. (2001). NF-κB: A key role in inflammatory diseases. J. Clin. Invest..

[61] Lopez-Posadas R., Ballester I., Abadia-Molina A.C., Suarez M.D., Zarzuelo A., Martinez-Augustin O., Sanchez de Medina F. (2008). Effect of flavonoids on rat splenocytes, a structure-activity relationship study. Biochem. Pharmacol..

[62] Kim H.P., Son K.H., Chang H.W., Kang S.S. (2004). Anti-inflammatory plant flavonoids and cellular action mechanisms. J. Pharmacol. Sci..

[63] Havsteen B.H. (2002). The biochemistry and medical significance of the flavonoids. Pharmacol. Ther..

[64] Cazarolli L.H., Zanatta L., Alberton E.H., Figueiredo M.S., Folador P., Damazio R.G., Pizzolatti M.G., Silva F.R. (2008). Flavonoids: Prospective drug candidates. Mini Rev. Med. Chem..

[65] Biesalski H.K. (2007). Polyphenols and inflammation: Basic interactions. Curr. Opin. Clin. Nutr. Metab. Care.

[66] Yoon J.H., Baek S.J. (2005). Molecular targets of dietary polyphenols with anti-inflammatory properties. Yonsei. Med. J..

[67] O’Leary K.A., de Pascual-Tereasa S., Needs P.W., Bao Y.P., O’Brien N.M., Williamson G. (2004). Effect of flavonoids and vitamin E on cyclooxygenase-2 (COX-2) transcription. Mutat. Res..

[68] Coutinho M.A.S., Muzitano M.F., Costa S.N.S. (2009). Flavonóides: Potenciais agentes terapêuticos para o processo inflamatório. Rev. Virtual Quím..

[69] Robak J., Gryglewski R.J. (1996). Bioactivity of flavonoids. Pol J. Pharmacol..

[70] Kim H.P., Mani I., Iversen L., Ziboh V.A. (1998). Effects of naturally-occurring flavonoids and biflavonoids on epidermal cyclooxygenase and lipoxygenase from guinea-pigs. Prostaglandins Leukot. Essent. Fatty Acids.

[71] Hirano T., Higa S., Arimitsu J., Naka T., Shima Y., Ohshima S., Fujimoto M., Yamadori T. Kawase, Tanaka T. (2004). Flavonoids such as luteolin, fisetin and apigenin are inhibitors of interleukin-4 and interleukin-13 production by activated human basophils. Int. Arch. Allergy Immunol..

[72] Lattig J., Bohl M., Fischer P., Tischer S., Tietbohl C., Menschikowski M., Gutzeit H.O., Metz P., Pisabarro M.T. (2007). Mechanism of inhibition of human secretory phospholipase A2 by flavonoids: Rationale for lead design. J. Comput. Aided Mol. Des..

[73] Rotelli A.E., Guardia T., Juarez A.O., de la Rocha N.E., Pelzer L.E. (2003). Comparative study of flavonoids in experimental models of inflammation. Pharmacol. Res..

[74] Moroney M.A., Alcaraz M.J., Forder R.A., Carey F., Hoult J.R. (1988). Selectivity of neutrophil 5-lipoxygenase and cyclo-oxygenase inhibition by an anti-inflammatory flavonoid glycoside and related aglycone flavonoids. J. Pharm. Pharmacol..

[75] Hollman P.C., Katan M.B. (1999). Dietary flavonoids: Intake, health effects and bioavailability. Food Chem. Toxicol..

[76] Bae E.A., Han M.J., Lee M., Kim D.H. (2000). *In vitro* inhibitory effect of some flavonoids on rotavirus infectivity. Biol. Pharm. Bull..

[77] Lim H.J., Jin H.G., Woo E.R., Lee S.K., Kim H.P. (2013). The root barks of Morus alba and the flavonoid constituents inhibit airway inflammation. J. Ethnopharmacol..

[78] Ferlay J., Shin H.R., Bray F., Forman D., Mathers C., Parkin D.M. (2010). Estimates of worldwide burden of cancer in 2008: GLOBOCAN 2008. Int. J. Cancer.

[79] (2014). From the Global Strategy for the Diagnosis, Management and Prevention of COPD, Global Initiative for Chronic Obstructive Lung Disease (GOLD). http://www.goldcopd.org/.

[80] MacNee W., Rahman I. (2001). Is oxidative stress central to the pathogenesis of chronic obstructive pulmonary disease?. Trends Mol. Med..

[81] Nowak D., Kasielski M., Antczak A., Pietras T., Bialasiewicz P. (1999). Increased content of thiobarbituric acid-reactive substances and hydrogen peroxide in the expired breath condensate of patients with stable chronic obstructive pulmonary disease: No significant effect of cigarette smoking. Respir. Med..

[82] Lagente V., Manoury B., Nenan S., le Quement C., Martin-Chouly C., Boichot E. (2005). Role of matrix metalloproteinases in the development of airway inflammation and remodeling. Braz. J. Med. Biol. Res..

[83] Demedts I.K., Demoor T., Bracke K.R., Joos G.F., Brusselle G.G. (2006). Role of apoptosis in the pathogenesis of COPD and pulmonary emphysema. Respir. Res..

[84] Tabak C., Arts I.C., Smit H.A., Heederik D., Kromhout D. (2001). Chronic obstructive pulmonary disease and intake of catechins, flavonols, and flavones: The MORGEN Study. Am. J. Respir. Crit. Care Med..

[85] Weseler A.R., Geraets L., Moonen H.J.J., Manders R.J.F., van Loon L.J.C., Pennings H.-J., Wouters E.F.M., Bast A., Hageman G.J. (2009). Poly (ADP-ribose) polymerase-1-inhibiting Flavonoids Attenuate Cytokine Release in Blood from Male Patients with Chronic Obstructive Pulmonary Disease or Type 2 Diabetes. J. Nutr..

[86] Park H.K., Kim S.J., Kwon D.Y., Park J.H., Kim Y.C. (2010). Protective effect of quercetin against paraquat-induced lung injury in rats. Life Sci..

[87] Chirumbolo S. (2010). The role of quercetin, flavonols and flavones in modulating inflammatory cell function. Inflamm. Allergy Drug Targets.

[88] Hayashi Y., Matsushima M., Nakamura T., Shibasaki M., Hashimoto N., Imaizumi K., Shimokata K., Hasegawa Y., Kawabe T. (2012). Quercetin protects against pulmonary oxidant stress via heme oxygenase-1 induction in lung epithelial cells. Biochem. Biophys. Res. Commun..

[89] Li N., Li Q., Zhou X.D., Kolosov V.P., Perelman J.M. (2012). The effect of quercetin on human neutrophil elastase-induced mucin5AC expression in human airway epithelial cells. Int. Immunopharmacol..

[90] Rakotomalala G., Agard C., Tonnerre P., Tesse A., Derbre S., Michalet S., Hamzaoui J., Rio M., Cario-Toumaniantz C., Richomme P. (2013). Extract from Mimosa pigra attenuates chronic experimental pulmonary hypertension. J. Ethnopharmacol..

[91] Johansson S., Goransson U., Luijendijk T., Backlund A., Claeson P., Bohlin L. (2002). A neutrophil multitarget functional bioassay to detect anti-inflammatory natural products. J. Nat. Prod..

[92] Kanashiro A., Souza J.G., Kabeya L.M., Azzolini A.E., Lucisano-Valim Y.M. (2007). Elastase release by stimulated neutrophils inhibited by flavonoids: Importance of the catechol group. Z Naturforsch. C.

[93] Walker E.H., Pacold M.E., Perisic O., Stephens L., Hawkins P.T., Wymann M.P., Williams R.L. (2000). Structural determinants of phosphoinositide 3-kinase inhibition by wortmannin, LY294002, quercetin, myricetin, and staurosporine. Mol. Cell.

[94] Ganesan S., Faris A.N., Comstock A.T., Chattoraj S.S., Chattoraj A., Burgess J.R., Curtis J.L., Martinez F.J., Zick S., Hershenson M.B. (2010). Quercetin prevents progression of disease in elastase/LPS-exposed mice by negatively regulating MMP expression. Respir. Res..

[95] Guan Y., Li F.F., Hong L., Yan X.F., Tan G.L., He J.S., Dong X.W., Bao M.J., Xie Q.M. (2011). Protective effects of liquiritin apioside on cigarette smoke-induced lung epithelial cell injury. Fundam. Clin. Pharmacol..

[96] Fanelli V., Vlachou A., Ghannadian S., Simonetti U., Slutsky A.S., Zhang H. (2013). Acute respiratory distress syndrome: New definition, current and future therapeutic options. J. Thorac. Dis..

[97] Chen J., Wang J.B., Yu C.H., Chen L.Q., Xu P., Yu W.Y. (2013). Total flavonoids of Mosla scabra leaves attenuates lipopolysaccharide-induced acute lung injury via down-regulation of inflammatory signaling in mice. J. Ethnopharmacol..

[98] Tseng T.L., Chen M.F., Tsai M.J., Hsu Y.H., Chen C.P., Lee T.J. (2012). Oroxylin-A rescues LPS-induced acute lung injury via regulation of NF-kappaB signaling pathway in rodents. PLoS One.

[99] Kuo M.Y., Liao M.F., Chen F.L., Li Y.C., Yang M.L., Lin R.H., Kuan Y.H. (2011). Luteolin attenuates the pulmonary inflammatory response involves abilities of antioxidation and inhibition of MAPK and NFkappaB pathways in mice with endotoxin-induced acute lung injury. Food Chem. Toxicol..

[100] Chen X., Yang X., Liu T., Guan M., Feng X., Dong W., Chu X., Liu J., Tian X., Ci X. (2012). Kaempferol regulates MAPKs and NF-kappaB signaling pathways to attenuate LPS-induced acute lung injury in mice. Int. Immunopharmacol..

[101] Gong J.H., Shin D., Han S.Y., Park S.H., Kang M.K., Kim J.L., Kang Y.H. (2013). Blockade of airway inflammation by kaempferol via disturbing Tyk-STAT signaling in airway epithelial Cells and in asthmatic mice. Evid. Based Complement. Alternat. Med..

[102] Soromou L.W., Chu X., Jiang L., Wei M., Huo M., Chen N., Guan S., Yang X., Chen C., Feng H. (2012). *In vitro* and *in vivo* protection provided by pinocembrin against lipopolysaccharide-induced inflammatory responses. Int. Immunopharmacol..

[103] Li H.B., Chen F. (2005). Isolation and purification of baicalein, wogonin and oroxylin A from the medicinal plant Scutellaria baicalensis by high-speed counter-current chromatography. J. Chromatogr. A.

[104] Chen Y., Nie Y.C., Luo Y.L., Lin F., Zheng Y.F., Cheng G.H., Wu H., Zhang K.J., Su W.W., Shen J.G. (2013). Protective effects of naringin against paraquat-induced acute lung injury and pulmonary fibrosis in mice. Food Chem. Toxicol..

[105] Bateman E.D., Hurd S.S., Barnes P.J., Bousquet J., Drazen J.M., FitzGerald M., Gibson P., Ohta K., O’Byrne P., Pedersen S.E. (2008). Global strategy for asthma management and prevention: GINA executive summary. Eur. Respir. J..

[106] Masoli M., Fabian D., Holt S., Beasley R. (2004). The global burden of asthma: Executive summary of the GINA dissemination committee report. Allergy.

[107] Devereux G., Seaton A. (2005). Diet as a risk factor for atopy and asthma. J. Allergy Clin. Immunol..

[108] Knekt P., Kumpulainen J., Jarvinen R., Rissanen H., Heliovaara M., Reunanen A., Hakulinen T., Aromaa A. (2002). Flavonoid intake and risk of chronic diseases. Am. J. Clin. Nutr..

[109] Fewtrell C.M., Gomperts B.D. (1977). Effect of flavone inhibitors of transport ATPases on histamine secretion from rat mast cells. Nature.

[110] Middleton E., Drzewiecki G., Krishnarao D. (1981). Quercetin: An inhibitor of antigen-induced human basophil histamine release. J. Immunol..

[111] Cheong H., Ryu S.Y., Oak M.H., Cheon S.H., Yoo G.S., Kim K.M. (1998). Studies of structure activity relationship of flavonoids for the anti-allergic actions. Arch. Pharm. Res..

[112] Townsend E.A., Emala C.W. (2013). Quercetin acutely relaxes airway smooth muscle and potentiates beta-agonist-induced relaxation via dual phosphodiesterase inhibition of PLCbeta and PDE4. Am. J. Physiol. Lung Cell Mol. Physiol..

[113] Jung C.H., Lee J.Y., Park J.H., Cho B.J., Sim S.S., Kim C.J. (2010). Flavonols attenuate the immediate and late-phase asthmatic responses to aerosolized-ovalbumin exposure in the conscious guinea pig. Fitoterapia.

[114] Kimata M., Shichijo M., Miura T., Serizawa I., Inagaki N., Nagai H. (2000). Effects of luteolin, quercetin and baicalein on immunoglobulin E-mediated mediator release from human cultured mast cells. Clin. Exp. Allergy.

[115] Kimata M., Inagaki N., Nagai H. (2000). Effects of luteolin and other flavonoids on IgE-mediated allergic reactions. Planta Med..

[116] Nanua S., Zick S.M., Andrade J.E., Sajjan U.S., Burgess J.R., Lukacs N.W., Hershenson M.B. (2006). Quercetin blocks airway epithelial cell chemokine expression. Am. J. Respir. Cell Mol. Biol..

[117] Rogerio A.P., Kanashiro A., Fontanari C., da Silva E.V., Lucisano-Valim Y.M., Soares E.G., Faccioli L.H. (2007). Anti-inflammatory activity of quercetin and isoquercitrin in experimental murine allergic asthma. Inflamm. Res..

[118] Higa S., Hirano T., Kotani M., Matsumoto M., Fujita A., Suemura M., Kawase I., Tanaka T. (2003). Fisetin, a flavonol, inhibits TH2-type cytokine production by activated human basophils. J. Allergy Clin. Immunol..

[119] Hirano T., Higa S., Arimitsu J., Naka T., Ogata A., Shima Y., Fujimoto M., Yamadori T., Ohkawara T., Kuwabara Y. (2006). uteolin, a flavonoid, inhibits AP-1 activation by basophils. Biochem. Biophys. Res. Commun..

[120] Mastuda H., Morikawa T., Ueda K., Managi H., Yoshikawa M. (2002). Structural requirements of flavonoids for inhibition of antigen-Induced degranulation, TNF-alpha and IL-4 production from RBL-2H3 cells. Bioorg. Med. Chem..

[121] Jayaprakasam B., Doddaga S., Wang R., Holmes D., Goldfarb J., Li X.M. (2009). Licorice flavonoids inhibit eotaxin-1 secretion by human fetal lung fibroblasts *in vitro*. J. Agric. Food Chem..

[122] Duan W., Kuo I.C., Selvarajan S., Chua K.Y., Bay B.H., Wong W.S. (2003). Antiinflammatory effects of genistein, a tyrosine kinase inhibitor, on a guinea pig model of asthma. Am. J. Respir. Crit. Care Med..

[123] Bao Z.S., Hong L., Guan Y., Dong X.W., Zheng H.S., Tan G.L., Xie Q.M. (2011). Inhibition of airway inflammation, hyperresponsiveness and remodeling by soy isoflavone in a murine model of allergic asthma. Int. Immunopharmacol..

[124] Hernandez V., Recio M.C., Manez S., Giner R.M., Rios J.L. (2007). Effects of naturally occurring dihydroflavonols from Inula viscosa on inflammation and enzymes involved in the arachidonic acid metabolism. Life Sci..

[125] Taguchi L., Pinheiro N., Olivo C., Grecco S., Choqueta-Toledo A., Lopes F., Martins M., Tiberio I., Lago J., Prado C. (2012). 5,6,7-Trihydroxy-4-methoxy.-flavanone from Baccharis. retusa. (Asteraceae.) attenuates elastase-induced emphysema in mice. Am. J. Respir. Crit. Care. Med..

[126] Li L., Bao H., Wu J., Duan X., Liu B., Sun J., Gong W., Lv Y., Zhang H., Luo Q. (2012). Baicalin is anti-inflammatory in cigarette smoke-induced inflammatory models *in vivo* and *in vitro*: A possible role for HDAC2 activity. Int. Immunopharmacol..

[127] Kalhan R., Smith L.J., Nlend M.C., Nair A., Hixon J.L., Sporn P.H. (2008). A mechanism of benefit of soy genistein in asthma: Inhibition of eosinophil p38-dependent leukotriene synthesis. Clin. Exp. Allergy.

[128] Smith L.J., Holbrook J.T., Wise R., Blumenthal M., Dozor A.J., Mastronarde J., Williams L. (2004). Dietary intake of soy genistein is associated with lung function in patients with asthma. J. Asthma.

